# Unlocking the human inner ear for therapeutic intervention

**DOI:** 10.1038/s41598-022-22203-2

**Published:** 2022-11-08

**Authors:** Hao Li, Sumit Agrawal, Seyed Alireza Rohani, Ning Zhu, Daniela I. Cacciabue, Marcelo N. Rivolta, Douglas E. H. Hartley, Dan Jiang, Hanif M. Ladak, Gerard M. O’Donoghue, Helge Rask-Andersen

**Affiliations:** 1 Department of Surgical Sciences, Otorhinolaryngology and Head and Neck Surgery, Uppsala University, Uppsala, Sweden; 2 Department of Otolaryngology-Head and Neck Surgery, Western University, London, ON Canada; 3 Department of Medical Biophysics, Western University, London, ON Canada; 4 Department of Electrical and Computer Engineering, Western University, London, ON Canada; 5 School of Biomedical Engineering, Western University, London, ON Canada; 6 Canadian Light Source, Saskatoon, Canada; 7 Department of Chemical and Biological Engineering, College of Engineering, University of Saskatchewan, Saskatoon, Canada; 8 School of Biosciences, Centre for Stem Cell Biology, University of Sheffield, Sheffield, UK; 9 National Institute for Health Research (NIHR) Nottingham Biomedical Research Centre, Nottingham, UK; 10 Queens Medical Centre, Nottingham University Hospitals NHS Trust, Nottingham, UK; 11 Otology and Hearing Group, Division of Clinical Neuroscience, School of Medicine, University of Nottingham, Nottingham, UK; 12 Centre for Craniofacial and Regenerative Biology, King’s College London, London, UK; 13 Hearing Implant Centre, Guy’s and St. Thomas’ NHS Foundation Trust, London, UK

**Keywords:** Sensory systems, Translational research, Anatomy, Medical research

## Abstract

The human inner ear contains minute three-dimensional neurosensory structures that are deeply embedded within the skull base, rendering them relatively inaccessible to regenerative therapies for hearing loss. Here we provide a detailed characterisation of the functional architecture of the space that hosts the cell bodies of the auditory nerve to make them safely accessible for the first time for therapeutic intervention. We used synchrotron phase-contrast imaging which offers the required microscopic soft-tissue contrast definition while simultaneously displaying precise bony anatomic detail. Using volume-rendering software we constructed highly accurate 3-dimensional representations of the inner ear. The cell bodies are arranged in a bony helical canal that spirals from the base of the cochlea to its apex; the canal volume is 1.6 μL but with a diffusion potential of 15 μL. Modelling data from 10 temporal bones enabled definition of a safe trajectory for therapeutic access while preserving the cochlea’s internal architecture. We validated the approach through surgical simulation, anatomical dissection and micro-radiographic analysis. These findings will facilitate future clinical trials of novel therapeutic interventions to restore hearing.

## Introduction

By 2050, disabling hearing loss due to damage to the sensorineural structures of the inner ear will affect over 700 million individuals globally with major health, economic and societal implications^1^. Yet no biological treatment exists for this disabling condition. Auditory prostheses, such as hearing aids and cochlear implants, represent the mainstay of current management but have considerable functional limitations. The greatest challenge to-day is to find a curative treatment for hearing loss through restoration of the neurosensory substrates that underpin our ability to hear. This challenge is formidable as the human cochlea is a highly specialised post-mitotic organ with highly restricted proliferative and regenerative capabilities. The cochlea’s sensory receptors comprise vibration-sensitive hair cells, their synapses and the neurones that subserve them. The system is exquisitely finetuned and can respond within millionths of a second to displacement of atomic dimensions. These neurosensory structures are fragile and may be permanently lost due to genetic and environmental factors and are particularly susceptible to the ageing process. The hearing loss may be due to a loss of hair cells^2^, synaptic dysfunction^3^ or to depletion of the neural population within the cochlea^4^ as well as a dysfunction of the stria vascularis and fibrocyte network^5^. The alarming rise in prevalence of hearing loss, now affecting 7% of the world’s population, is a major stimulus to exploit the potential of regenerative medicine in this field^6^.

Hampering progress has been the inaccessibility of the human cochlea which lies in the skull base deeply encased in the hardest bone in the human body. Yet, once accessed, the cochlea promises to be a receptive organ for neurosensory regeneration: the neurosensory cells are relatively few in number and its minute fluid compartments (with a total volume around 200 μL) are tightly confined with a negligible circulation which should facilitate biological effectiveness and restricted biodistribution, minimising off-target effects. In addition, cochlear tissues are relatively immune-privileged being protected by the blood-labyrinth barrier thus dampening the inflammatory rejection process. Recent elaboration of a range of molecular mechanisms responsible for inner ear dysfunction have opened a vista of opportunities for a range of novel therapeutic approaches to hearing loss including small molecules, gene and cell therapies^7^. Central to success, however, is the ability to deliver these agents safely and precisely to their target structures within the relatively impenetrable human cochlea. Encouraging evidence from non-human mammalian studies have shown that targeted administration of human stem cell-derived otic progenitors can result in hearing restoration^8^. To exert their therapeutic effects, otic progenitors were delivered precisely to their target structures in the cochlear neural space of gerbils (Supplementary Fig. 1). Until now, an equivalent access has not been available for the human cochlea. In this report we focus on the human spiral ganglion (HSG) cell population, aiming to create for the first time an accurate three-dimensional model, analysing its detailed functional cytoarchitecture and how it might be safely accessed for regenerative interventions in humans.

The structures of the cochlea, such as the basilar and Reissner’s membranes, are microscopic and well beyond the resolution of clinical imaging modalities. Although micro-computed tomography provides sufficient spatial resolution, contrast agents are necessary to discern soft tissues but may lead to tissue shrinkage^9^. Conventional histological techniques, have been susceptible to dissection, decalcification and staining artefacts. We thus used synchrotron radiation phase-contrast imaging (SR-PCI) to delineate these minute structures while leaving them in situ. Several synchrotron studies applied to the inner ear have been published recently^10–12^, but here we have greatly extended this work to define and validate a novel therapeutic corridor to the human inner ear. SR-PCI differs from conventional radiography in being able to allow a phase shifted beam to interact with the original beam to produce fringes that represent the structural and surface boundaries (edge enhancement) of a specimen. This phase-contrast imaging produces images with excellent soft-tissue and bone discrimination and made it possible to accurately image the detailed cytoarchitecture of the human inner ear, in particular to access the structures that harbour the cochlear neurons in the intact state without incurring artefacts that so compromised previous anatomical studies. The use of advanced computer vision tools enabled fine blood vessels to be imaged and the pathways of nerves to be tracked through to the bony core (or modiolus) of the cochlea. This level of detail subsequently informed the planning of a safe surgical approach for clinical application.

## Results

**Figure 1:**
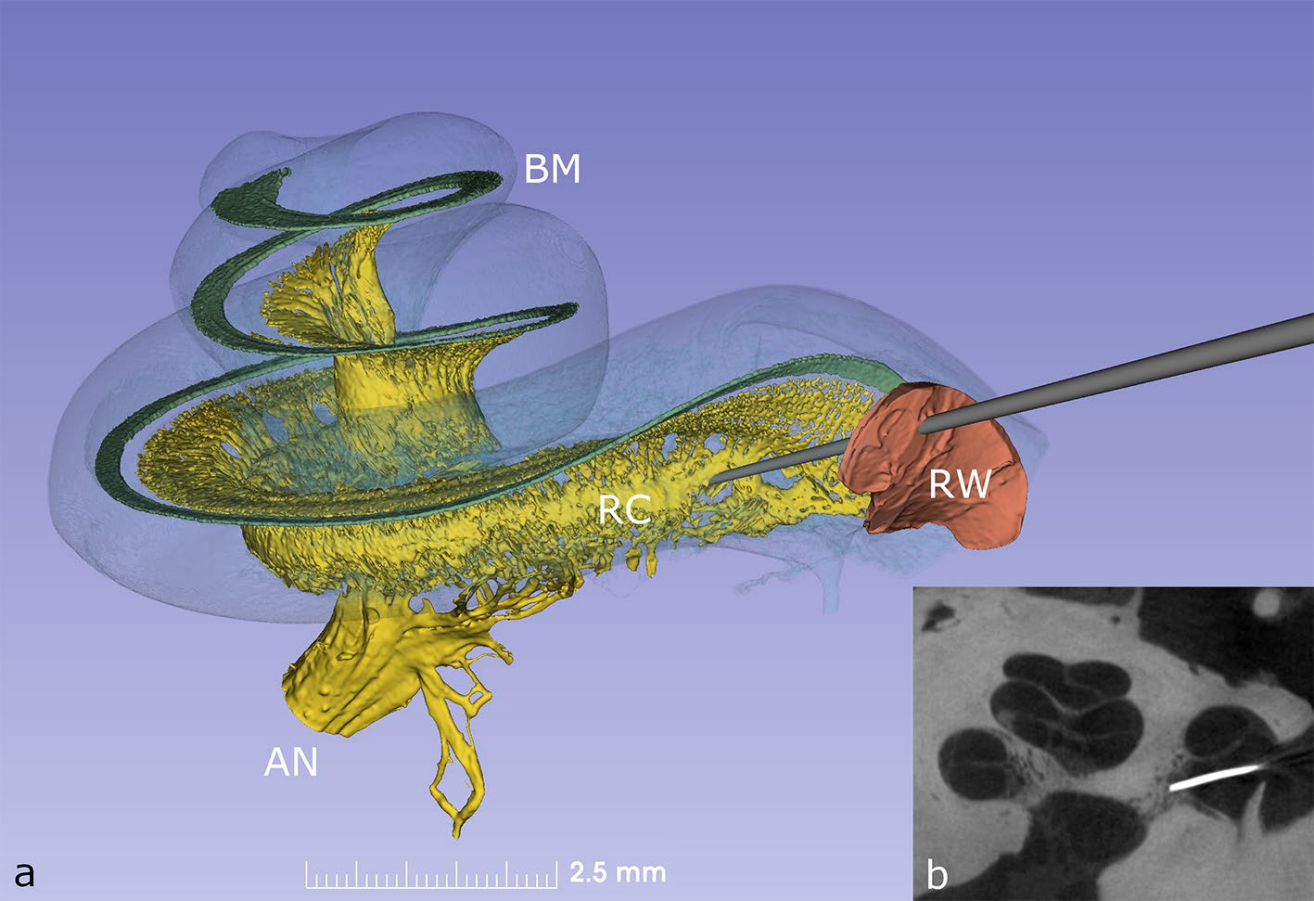
(**a**) Synchrotron phase contrast imaging (SR-PCI) with 3D orthographic rendering of an intact left human inner ear. The bony wall of the cochlea was made semi-transparent to permit visualization of the basilar membrane (BM), Rosenthal’s canal (RC) and auditory nerve (AN). The auditory nerve contains approx. 30,000 fibres and their cell bodies are located in a 14.5 mm long spiral bony canal called RC. From there, peripheral neurites spread out to innervate approx. 15,000 hair cells placed on the BM. A probe is shown penetrating the round window (RW) membrane to access the underlying RC. (**b**) Microradiograph taken following placement of a radio-opaque marker at the presumed site of RC on anatomical dissection; the image confirms precise targeting of RC during dissection.

Rosenthal’s canal (RC), which houses the cell bodies of the 30 to 35,000 human spiral ganglion (HSG) neurons of the auditory nerve, hugs the modiolus and extends from its base to near the cochlear apex (Fig. 1). The physical characteristics of RC are described in Table 1 (and Supplementary Table 1). Our analysis determined that it averages 14.57 mm in length (range 14.02–15.08 mm) and that it is covered by bone with a thickness of 28–56 µm which may be deficient in parts. The diameter of RC varies from 0.1 to 0.5 mm, being at its greatest towards the apex of the helix and has an average volume of 1.6 mm^3^ (Supplementary Fig. 2). The central projections of the auditory nerve traverse a virtual space containing cerebrospinal fluid as they exit the cochlea on their way to the brain-stem (Supplementary Fig. 3). We calculated this space to have a potential volume of 15 mm^3^ and as these nerve fibres are devoid of perineurium and may thus be receptive to cell or gene-based therapies.

**Table 1:** The length, diameter and volume of Rosenthal’s Canal (RC) as determined by synchrotron phase-contrast imaging in the 10 human temporal bones in the study.

Bone #	RC length (mm)	RC diameter (mm)			RC volume (mm^3^)
	Total	RC base	45°	90°	Total
1	14.02	0.133	0.291	0.435	1.182
2	14.73	0.100	0.366	0.498	1.376
3	14.28	0.109	0.395	0.464	1.087
4	14.61	0.060	0.442	0.430	1.392
5	14.22	0.116	0.432	0.495	1.668
6	15.08	0.143	0.382	0.464	1.746
7	14.91	0.103	0.482	0.590	1.246
8	14.58	0.109	0.494	0.567	2.315
9	14.27	0.122	0.468	0.444	2.012
10	14.98	0.096	0.519	0.637	2.150
Average	14.568	0.109	0.427	0.502	1.617
SD	0.36058	0.023	0.069	0.072	0.430

The diameter of the RC is larger with increasing distance from the base of the cochlea.

**Figure 2:**
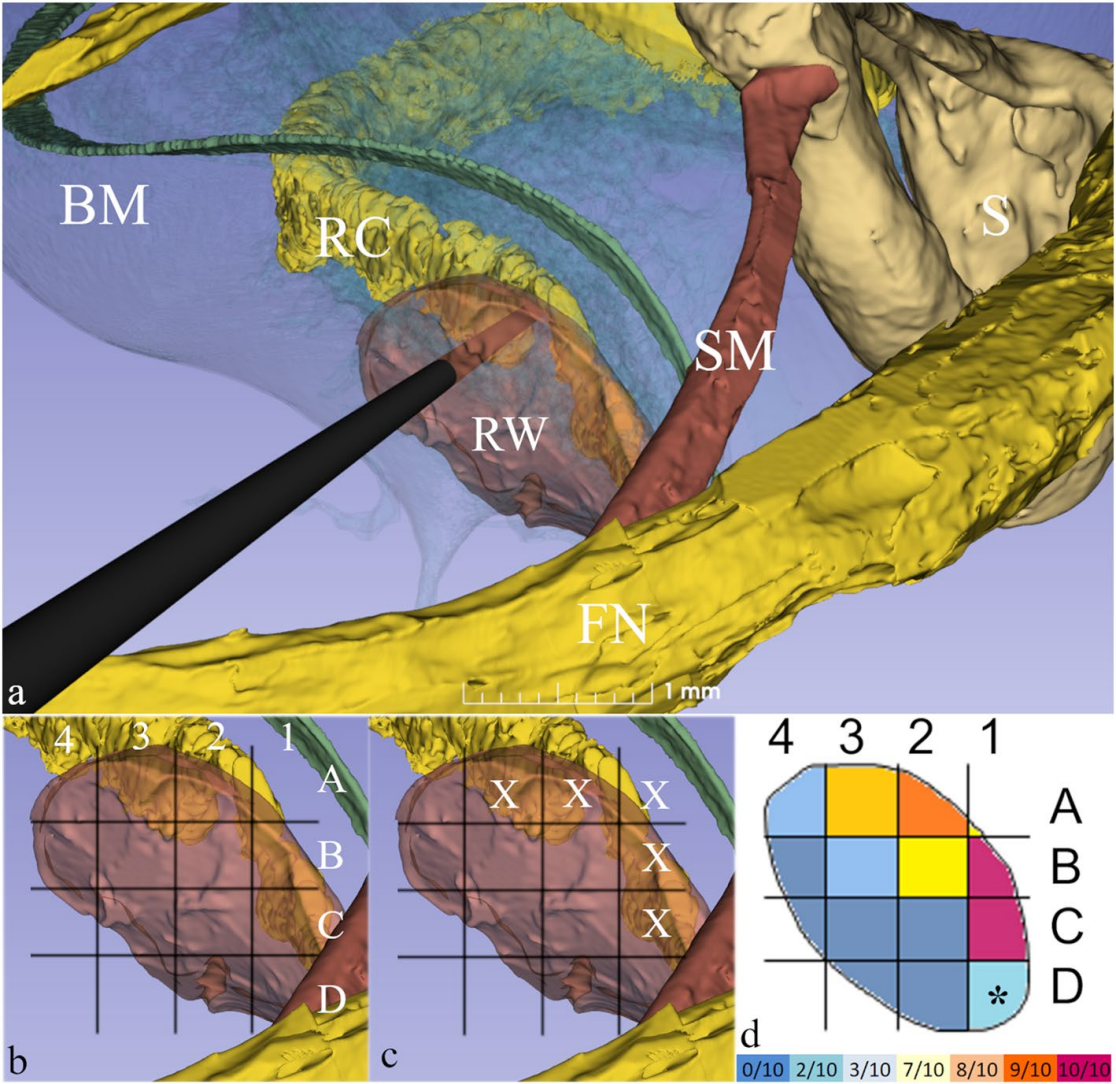
(**a**) The surgical approach to left Rosenthal’s Canal (RC) passing through the mastoid bone behind the ear. Note the structures of surgical interest: the facial nerve (FN), the stapes (S) and stapedius muscle (SM) and round window membrane (RW). A surgical trephine is seen penetrating the RW to reach RC located just deep to it. (**b**) A 4 × 4 dynamic grid was applied to 10 RW membranes and was adjusted to membrane size for each specimen. (**c**) X denotes those grid units in closest anatomical proximity to RC in which a given penetration had at least an 80% chance of reaching RC (see Supplementary Table 2). (**d**) Using this data, a heat map was created for optimal targeting of RC behind the RW membrane. Highest probability of targeting the RC was through the superior mid-region of the RW membrane. At A2 and A3, the chance was 90% and 80% respectively. Higher values were noted in C1 and B1 (100%), but this region risks injury to the vestibulo-cochlear artery. *D1 was covered by stapedius muscle and was thus not evaluable. Colour bar at the bottom displays the actual frequencies of successfully targeting the RC.

Fortunately, at the base of the cochlea the RC lies in close proximity to the round window membrane (Fig. 1 and Supplementary Fig. 4), a structure that is easily identifiable during routine ear surgery and that can be readily penetrated or reflected to gain access to RC. We thus used our SR-PCI 3-dimensional models to define the round window to RC relationships and then undertook a series of surgical simulations, formulating a heat map to define the surgical trajectory that would lead directly to RC with the highest probability (Fig. 2). We found that by using a dynamic grid adjusted to the size of the round window that setting the point of penetration superiorly would reach target in 80% of cases without compromising cochlear blood supply which runs immediately adjacent (Supplementary Fig. 5 and Supplementary Table 2). Depending on the trajectory used, the contents of RC lay just 3–4 mm deep to the round window. Furthermore, to facilitate clinical translation, we ensured the trajectories we planned were consistent with instrumentation and approaches compatible with those used in routine ear surgery (Supplementary video).

In order to confirm the validity of our proposed access, we carried out a series of anatomical micro-dissections on human temporal bones. The aim was to determine if RC could be reached using surgical approaches through the mastoid bone with standard instrumentation. Six temporal bones were used, with surgeons marking the expected site of RC by positioning a radio-opaque metallic marker. The specimens were scanned prior to dissection (to exclude structural anomalies) as well as following placement of the marker. The bones were scanned with micro-computed tomography, following a protocol described elsewhere^13^. In 5 of the 6 temporal bones, the marker was either within the RC or immediately adjacent to it results that were in line with those predicted from our modelling data; in one bone the marker ended up fractionally below target (Supplementary Fig. 6). In none of the temporal bones was the microvasculature disrupted and no unintended damage to other anatomical structures within the ear was observed.

## Discussion

The inability to adequately image the fine structures of the human inner ear has been a major barrier to advance therapies for this complex end-organ. SR-PCI is proving to be transformative in displaying and evaluating these microscopic structures providing unprecedented visualisation of its in-situ cytoarchitecture. Traditional methods used to design surgical routes to the cochlea, even with operating microscopes, were marred by their inherent destructive nature. We have demonstrated, that 3-dimensional models based on SR-PCI data allow the conception of highly accurate intervention pathways which were subsequently validated by anatomical dissection and micro-radiographic imaging. These findings will greatly facilitate the accurate delivery of novel therapeutic agents to their target structures in the inner ear and will thus derisk future clinical trials. The application of SR-PCI to the auditory system also dovetails with an escalation of interest in regenerative inner ear therapies which hold considerable promise for addressing the growing health burden of hearing loss^14–16^. While considerable challenges remain in developing novel therapeutics for use in humans^17^, we believe that these developments herald a new era for the application of regenerative therapeutics to the inner ear. Although SR-PCI cannot be used in humans in vivo its translational value is immense through enabling precise modelling of the inner ear microstructures to guide therapeutic access. These models can be further enhanced by automatic segmentation and deep-learning networks to improve accuracy and support clinical application^22^.

## Material and methods

Ten adult human temporal bones were obtained with permission from the Body Bequeathal Program at Western University, London, Ontario, Canada in accordance with and approved by the Anatomy Act of Ontario and Western University’s Committee for Cadaveric Use in Research (approval #19062014). The imaging technique used in this study is the propagation-based X-ray phase-contrast imaging (PCI) method, which is also known as in-line PC and has previously been adopted by us to image the auditory system^18–20^. Compared to conventional X-ray absorption based imaging, in-line PCI uses X-ray refraction which highlights tissue boundaries within a sample. It can be used to image soft tissues which do not absorb X-rays sufficiently to distinguish tissue components based on image contrast. A spatially coherent source is needed for in-line PCI, hence synchrotron radiation is used in this work rather than a conventional X-ray source. The overall set-up for in-line PCI is similar to typical absorption based radiography in that it consists of a source, a sample, and a detector; however, the main difference is that the detector is placed further from the sample when using in-line PCI, and this gives rise to Fresnel fringes. In-line PCI is sensitive to changes in refractive index which leads to edge enhancement in images.

SR-PCI scanning was performed at the Bio-Medical Imaging and Therapy (BMIT) 05ID-2 beamline at the Canadian Light Source Inc. located in Saskatoon, SK, Canada. The X-ray photon energy was 42 keV, with sample-to-source distance of 57 m and sample-to-detector distance of 2 m. The detector had field of view of 36 mm × 9.5 mm and pixel size of 9 µm, and 3000 projections were collected over 180° rotation. The reconstruction was performed using the UFO platform (www.github.com/ufo-kit), which is an open-source platform. To perform the quantitative analysis and 3D visualization, phase-retrieval technique was used to convert the edge enhancement caused by fringes, to areal contrast using Paganin/TIE method^21^. The reconstructed slices were then imported to 3D Slicer (www.slicer.org) for visualization, segmentation and measurements^18–20^. Manual threshold painting was performed for most anatomical structures. Measurements of volumes and distances to adjacent critical structures was then undertaken and trajectory maps for future surgical approaches were designed. Image segmentation was driven by the need to survey the anatomical structures of clinical interest. Semi-automatic and manual segmentation tools, threshold painting, thresholding, tractography, and scissors tools were used to display the fine detail of the structures of interest.

### Ethics approval

Permission was obtained from the Body Bequeathal Program at Western University, London, Ontario, Canada in accordance with and approved by the Anatomy Act of Ontario and Western University’s Committee for Cadaveric Use in Research (approval #19062014).

## Supplementary Information

Supplementary Video 1.

Supplementary Information 1.

## Data Availability

All data generated or analysed during this study are included in this published article and its supplementary information files.
