# Supplementary material for: Unlocking the human inner ear for therapeutic intervention
Source: Sci Rep. 2022 Nov 8;12:18508. doi: 10.1038/s41598-022-22203-2 (PMC9643346; doi:10.1038/s41598-022-22203-2)
Supplement: Supplementary file 2 — Supplementary Information 1. [file 41598_2022_22203_MOESM2_ESM.docx]

|  | **From middle of RWM to (mm)** | | | **Modiolus bone thickness (mm)** | | |
| --- | --- | --- | --- | --- | --- | --- |
| **Bone #** | RC base | 45° | 90° | RC base | 45° | 90° |
| **1** | 0.879 | 3.800 | 5.560 | 0.036 | 0.036 | 0.034 |
| **2** | 1.280 | 3.570 | 5.600 | 0.033 | 0.030 | 0.041 |
| **3** | 0.822 | 3.350 | 5.620 | 0.023 | 0.019 | 0.047 |
| **4** | 1.170 | 4.130 | 6.610 | 0.029 | 0.027 | 0.053 |
| **5** | 1.000 | 3.870 | 6.710 | 0.025 | 0.035 | 0.036 |
| **6** | 0.857 | 3.650 | 5.630 | 0.036 | 0.028 | 0.051 |
| **7** | 1.160 | 3.770 | 6.150 | 0.026 | 0.039 | 0.095 |
| **8** | 1.100 | 3.960 | 5.990 | 0.032 | 0.047 | 0.036 |
| **9** | 1.370 | 4.100 | 6.140 | 0.023 | 0.050 | 0.091 |
| **10** | 1.250 | 4.300 | 6.660 | 0.016 | 0.030 | 0.081 |
| **Average** | 1.089 | 3.850 | 6.067 | 0.028 | 0.034 | 0.056 |
| **SD** | 0.192 | 0.285 | 0.465 | 0.007 | 0.009 | 0.024 |

Supplementary Table 1. Shows the distance (mm) from the puncture site on the round window membrane (RWM) to Rosenthal’s canal (RC) at 45 and 90 degrees of angulation. Modiolus bone thickness is shown overlying RC. The thickness is variable and the bone may be absent in parts.

|  | A1 | A2 | A3 | A4 | B1 | B2 | B3 | B4 | C1 | C2 | C3 | C4 | D1 | D2 | D3 | D4 |
| --- | --- | --- | --- | --- | --- | --- | --- | --- | --- | --- | --- | --- | --- | --- | --- | --- |
| 1 | x | x | x |  | x | x | x |  | x |  |  |  | x |  |  |  |
| 2 |  | x | x | x | x | x |  |  | x |  |  |  | x |  |  |  |
| 3 |  | x |  |  | x |  |  |  | x |  |  |  |  |  |  |  |
| 4 | x | x | x |  | x | x |  |  | x |  |  |  |  |  |  |  |
| 5 | x | x | x |  | x |  |  |  | x |  |  |  |  |  |  |  |
| 6 | x | x | x | x | x | x |  |  | x |  |  |  |  |  |  |  |
| 7 | x | x | x |  | x | x |  |  | x |  |  |  |  |  |  |  |
| 8 |  | x | x |  | x | x | x |  | x |  |  |  |  |  |  |  |
| 9 | x | x | x | x | x | x | x |  | x |  |  |  |  |  |  |  |
| 10 | x |  |  |  | x |  |  |  | x |  |  |  |  |  |  |  |
| Total | 7 | 9 | 8 | 3 | 10 | 7 | 3 | 0 | 10 | 0 | 0 | 0 | 2 | 0 | 0 | 0 |

Supplementary Table 2. Data used to construct the heat map was derived from penetrating the various grid units on the RW membrane on 10 human temporal bones. X denotes a positive ‘hit’ occurring when a given trajectory reached target (RC). Penetrations at A2 and A3 safely reached Rosenthal’s canal at least 80% of time whereas those at C1 risked vascular injury to the cochlea thus limiting clinical application.


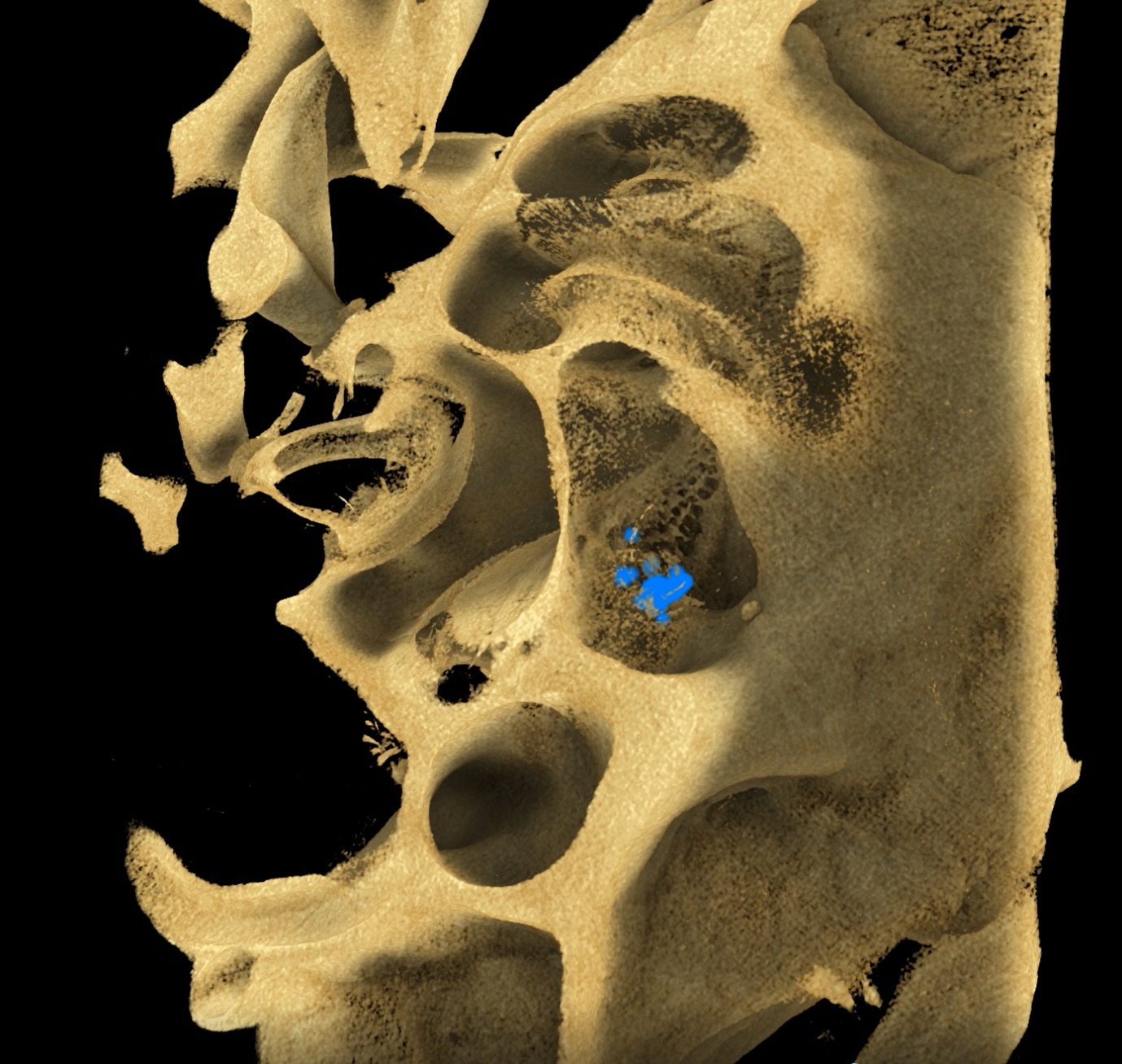


Supplementary Figure 1. Three-dimensional reconstruction of a micro-CT of a gerbil cochlea (left ear), showing glass beads (coloured in blue) delivered into the cochlear modiolus, using a similar approach to that employed in Chen et al 2012 (8) for the administration of otic progenitors. Briefly, this involved exposing the round window niche by opening a small hole in the bulla behind the pinna.The round window membrane was pierced using an Endo-Kflex dental file (#30) and a small hole drilled into the modiolous at the base of the cochlea. Glass beads were injected into the central modiolus using a stainless steel NanoFil syringe (WPI) with a 33G tungsten bevelled needle.


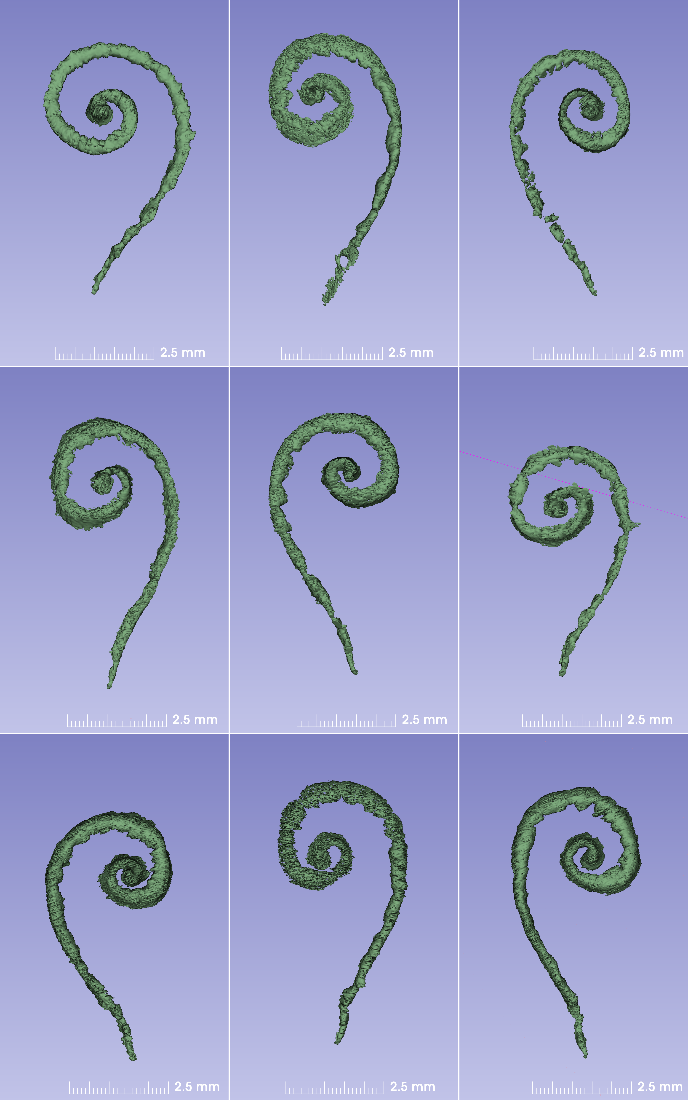


Supplementary Figure 2. Images of 9 Rosenthal canals (RCs) to illustrate the variability in shape and configuration of RC reflecting the differing sizes of the human cochlea. RC measures15 mm in length with an almost constant volume of approximately 1.6mm^3^.


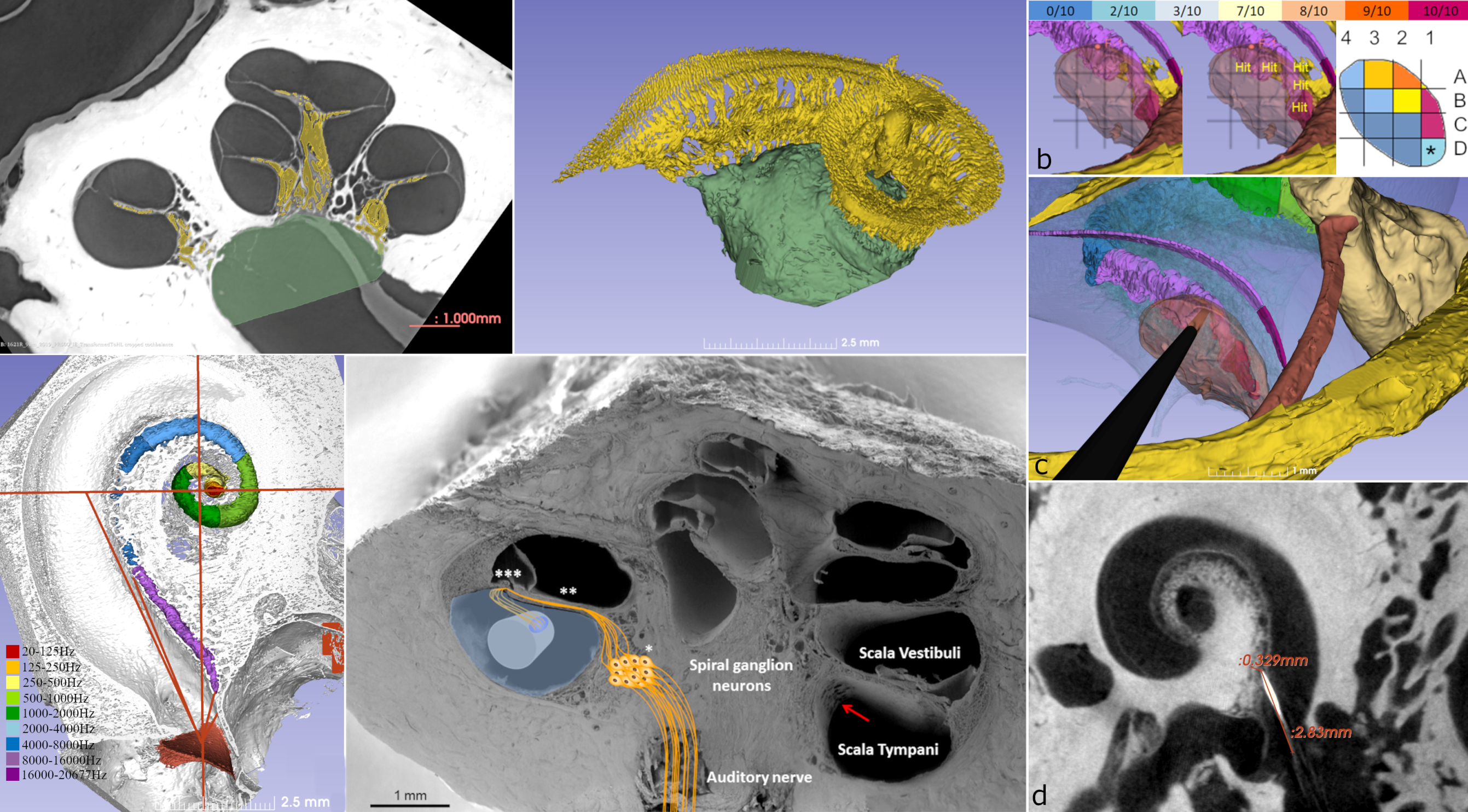


Supplementary Figure 3. 3-D reconstruction of synchrotron phase contrast images (SR-PCI) showing the fundus region of the human internal acoustic meatus (green) and the array of the auditory nerve fibres from the cochlear base to its apex (yellow). The auditory nerve fibres exit the cochlea through a bony cribriform area creating a potential space of 15 mm^3^ for therapeutic intervention.


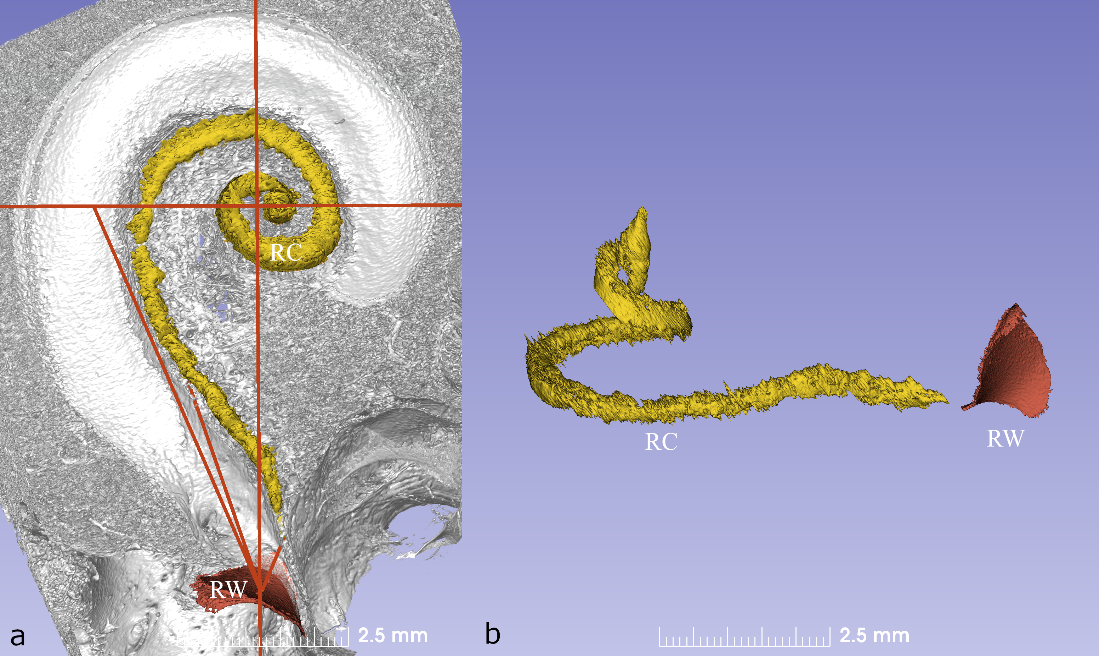


Supplementary Figure 4: Synchrotron phase contrast images (SR-PCI) showing the critical relationship between the left round window membrane (RW) and Rosenthal’s canal (RC) for therapeutic intervention. a. The RC is deeply encased in the cochlea’s bony central core and lying just deep to the RW. The angulation of instruments used to access RC is crucial to reaching target. b Showing the proximity of RW to RC which allows therapeutic access to spiral ganglion cells contained within RC.


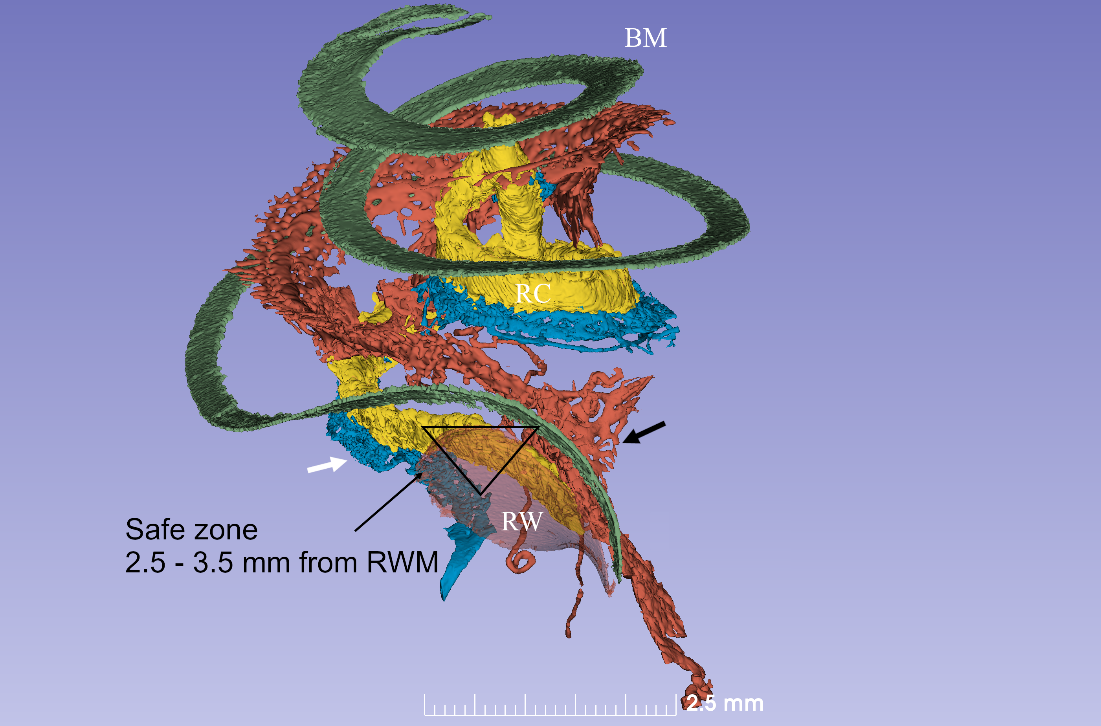


Supplementary Figure 5. The inferior cochlear vein (white arrow), the cochlear branch of the vestibulo-cochlear artery (black arrow) lie in close proximity to the round window membrane (RW). We identified a triangular area or ‘safe zone’ that would allow access to the left RC without compromising these structures, thus preserving cochlear function.


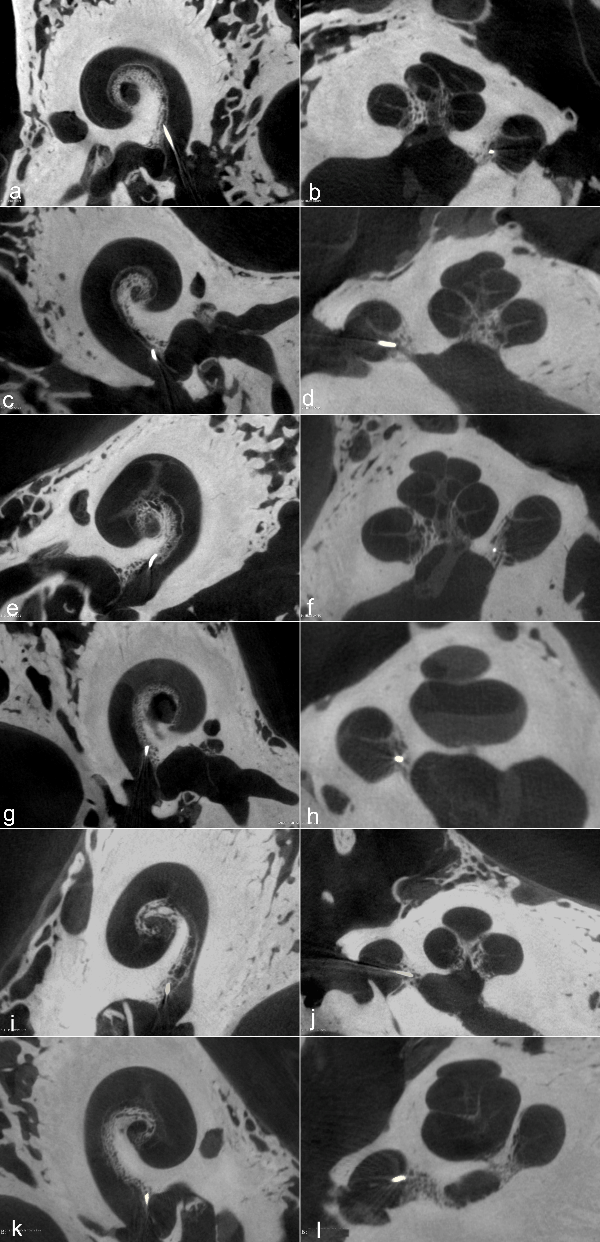


Supplementary Figure 6. Axial and coronal radio-micrographs of each of the 6 temporal bones used to validate the approach to Rosenthal’s canal (RC). A radio-opaque marker had been placed at the presumed location of RC on anatomical dissection. In 5 of the 6 temporal bones (a - j) target was reached; in one specimen (k - l) the marker was 0.3mm off target.

Supplementary Video

A video clip presenting the inner ear within a left temporal bone with schematic representation of the proposed therapeutic approach.

https://drive.google.com/file/d/1KVuuWjU4n946HXkHmlDyG7baRrJwCC2w/view?usp=sharing
